# The Experiences of Motor Skill Development in Children with Autism Spectrum Disorder (ASD) Reflected through Parental Responses

**DOI:** 10.3390/children11101238

**Published:** 2024-10-14

**Authors:** Anetta Müller, Éva Bácsné Bába, Peter Židek, Attila Lengyel, Judit Herpainé Lakó, Nóra Laoues-Czimbalmos, Anikó Molnár, Eszter Boda

**Affiliations:** 1 Department of Pedagogy, Faculty of Education, Selye János University, 3322 Komarno, Slovakia; 2 Institute of Sport Economics and Management, Faculty of Economics and Business, University of Debrecen, 4032 Debrecen, Hungary; bacsne.baba.eva@econ.unideb.hu (É.B.B.); molnar.aniko@econ.unideb.hu (A.M.); 3 Primary and Pre-School Education, Faculty of Education, Selye János University, 3322 Komarno, Slovakia; zidekp@ujs.sk; 4 Coordination and Research Centre for Social Sciences, Faculty of Economics and Business, University of Debrecen, 4032 Debrecen, Hungary; lengyel.attila@econ.unideb.hu; 5 Institute of Sport Science, Eszterházy Károly Catholic University, 3300 Eger, Hungary; lako.judit@uni-eszterhazy.hu (J.H.L.); boda.eszter@uni-eszterhazy.hu (E.B.); 6 Faculty of Child Education and Special Education, Department of Art and Health Education, University of Debrecen, 4032 Debrecen, Hungary; laoues.nora@ped.unideb.hu

**Keywords:** autism spectrum disorder, movement development, parental perspectives, individualized interventions, motor skills, communication skills, social skills, qualitative study

## Abstract

**Background/Objectives**: Understanding movement development in children with autism spectrum disorder (ASD) is critical for creating effective intervention strategies. This study aims to explore parental perspectives on the movement development of children with ASD, focusing on identifying common challenges and successful strategies. The objective is to analyze the experiences of parents to highlight the most effective approaches to support motor, communication, and social skills development in these children. **Methods**: Using a qualitative approach, we conducted in-depth interviews with parents of children with ASD. The data were analyzed using open and selective coding to identify key themes related to movement development challenges and strategies. The analysis included cross-referencing with the existing literature to support parental insights. **Results**: This study identified several key themes, including communication barriers, social interaction difficulties, and the importance of personalized movement programs. Parents reported significant challenges in understanding and addressing their children’s movement needs, particularly in group settings. However, activities such as water programs, music and dance, and animal-assisted therapies were found to be highly engaging. Motivation was a critical factor, with rewards and active parental involvement enhancing participation. **Conclusions**: This study highlights the crucial role of a supportive environment, including professional guidance and family support, in the success of movement development programs.

## 1. Introduction

Autism spectrum disorder (ASD) is one of the most common complex developmental disorders, with symptoms appearing in early childhood. According to current medical understanding, this condition is not curable [1,2]. Following the turn of the millennium, autism began to be referred to as autism spectrum disorder, emphasizing that symptoms can appear anywhere between the two extremes of the spectrum, from very severe forms to milder variants. Characteristics of individuals diagnosed with ASD include difficulties in social communication and social interaction and deficits in these abilities compared to typically developing individuals. However, there can be significant differences in the abilities of children and adults with ASD depending on where they are on the spectrum. Manifestations of autistic symptoms range from behaviors that cause significant disruption in daily life to behaviors that are often difficult to distinguish from typically developing peers [3].

The limitations in communication skills and the ability to form social relationships and interactions in autistic children can have very serious consequences in the areas of social, emotional, cognitive, and motor development alike. Children are social beings who need the presence of peers and shared interactions, games, and various activities being conducted with them, which also motivates them [4]. According to a study by Bukowski et al. [4], peer relationships significantly contribute to the development of social and emotional skills. Children learn how to initiate and maintain social interactions, as well as acquire skills such as conflict resolution, compromise, and cooperation. For young children, social relationships are particularly important as they help develop communication and social skills and emotional and cognitive development, as well as self-confidence and self-esteem. Additionally, they help in understanding cultural and social norms. These skills are fundamental for children’s overall development and for establishing competencies necessary for their later life [5]. Research has shown that due to various individual, socioeconomic and environmental factors, children with disabilities show lower participation rates in social activities compared to their typically developing peers [6,7,8]. According to the authors’ results, various individual, socioeconomic and environmental factors hinder these children’s active participation, contributing to their social isolation and a deterioration of their quality of life. Laoues’s work on the barriers experienced by children with disabilities in leisure and sports activities identifies several key obstacles. These barriers include physical limitations, lack of accessibility, social exclusion, and economic constraints [9,10].

The reported prevalence of autism spectrum disorder (ASD) varies significantly across countries, primarily due to differences in diagnostic practices, awareness, and healthcare system capabilities [11]. Global estimates suggest that ASD affects approximately 1–2% of the world’s population [12], translating to roughly 78–156 million individuals. However, this figure likely underestimates the true prevalence, as many cases remain undiagnosed, particularly in regions with limited resources and awareness.

Countries with more advanced healthcare and educational systems tend to report higher rates of ASD. This does not necessarily indicate a higher actual prevalence in these nations but rather reflects their enhanced ability to identify and diagnose cases [13]. For instance, the United Kingdom reports one of the highest rates globally at 1–1.5% of the population. The United States Centers for Disease Control and Prevention (CDC) estimates a prevalence of 1 in 36 children (2.8%) as of 2023 [14]. Other countries with reportedly high rates include Japan, Sweden, and Canada.

These figures are more indicative of robust diagnostic capabilities and greater public awareness rather than an inherently higher occurrence of ASD in these populations. A systematic review by [15] found that ASD prevalence estimates varied widely across countries, ranging from 0.013% to 2.9%, with much of this variation attributed to methodological differences in case identification and reporting.

It is crucial to interpret these statistics within the context of each country’s healthcare system, diagnostic criteria, and reporting mechanisms. As the global understanding of ASD improves and diagnostic practices become more standardized, we may see changes in reported prevalence rates across different regions, potentially revealing a more accurate picture of ASD’s global distribution.

In Canada, it is estimated that 1 in every 66 people between the ages of 5 and 17 has an ASD diagnosis [16]. In the United States, between 2010 and 2012, ASD was detected as 1 in 68 8-year-old children, and by 2014, it was 1 in 59 children [17].

In Hungary, several studies confirm the increasing trends in the number of people living with autism. According to the 2016 KSH microcensus analysis, which examined the proportion of people with disabilities between 2011 and 2016, in 2011, out of 6.2% of people with disabilities, nearly half a million people, only 1.04% identified themselves as having ASD. In terms of gender distribution, a higher prevalence was observed among males, with 5120 males and 894 females. In the 2016 KSH microcensus, 4.3% of the Hungarian population reported living with a disability, which was less than the results from the 2011 data, but the number of autistic individuals increased by more than 70%. The majority of autistic individuals in this case were also male, similar to the 2011 results [18].

An increasing number of autistic individuals can also be detected in the child population, similar to international trends, as in Hungary in the 2016/17 academic year, where 0.39% of students in public education institutions had ASD, which represented a significant increase compared to the 0.09% data in 2009 [19]. According to data recorded by the Hungarian Central Statistical Office since 2001, while in 2013, there were 3319 children living with autism spectrum disorder, by 2023, this number had increased to 9737 [20].

### Literature Review

Among the defining characteristics of ASD, we can name atypical social development, which includes language deficit and communication problems [3]. Frequently mentioned challenges include coordinating speech and eye contact and even a lack of genuine interest in others’ thoughts, as well as difficulties in receiving and conveying non-verbal messages (body language, the appropriate use of gestures, posture, and body language) [21,22,23,24].

Compared to the communication and social areas, research in the motor domain among children and young people with ASD is relatively underrepresented [25]. Compared to their typically developing peers and children with other developmental disorders, children with ASD have much lower social and leisure activity and participate in fewer activities, and the activities they do participate in are less varied [26,27,28]. International research on school-age children and adolescents with ASD has found that they have low participation in various activities at home or in school tasks and activities and even less in community activities [29,30]. Low participation in out-of-school leisure programs and activities, shared hobby groups, social relationships, and various recreational sports has also been proven [31,32]. However, an international study highlights the role of outdoor leisure and recreation programs among autistic children, as leisure sports practiced in nature also function as therapeutic tools suitable for improving the balance, mobility, and coordination skills of children with ASD, improving their physical health [33].

In the case of children with ASD, research and development strategies focusing on deficiencies in social skills and their development dominate [34,35], but more and more research is also directed towards motor skill deficits, uncoordinated movement, and the lack of balance [36,37,38]. Today, significant fine and gross motor difficulties, as well as coordination and balance problems, have been documented in various studies, and interventions also focus on these areas [39,40,41]. In the research of Pusponegoro and colleagues [42], some level of motor disorder was found in 79% of children with ASD, confirming the motor involvement experienced in the vast majority of autistic individuals.

Motor developmental delays occurring in early life stages were identified through retrospective video analyses [43]. Not only motor delay but also a slower rate of motor development has been confirmed, meaning that the motor developmental pace of children with ASD is slower than that of their typically developing peers [40]. During infant motor development, various natural movements and gross motor skills (rolling, crawling, rolling, sitting, standing, and walking) follow specific developmental milestones, and there is an optimal period for their formation. If the first appearance of these movement forms is delayed or appears on time but then cannot be produced, or if the formation of general coordination patterns only appears belatedly, these signs may indicate motor deficits of autism spectrum disorder. Several studies confirm the motor impairment observable in the early life stage and its diagnosable signs and therefore draw attention to the possibilities of early diagnosis and intervention [44,45,46,47].

Motor research has also supported that the motor difference between autistic and typically developing children’s motor skills can be observed as early as preschool age, and as time progresses, the difference in children’s motor skills becomes increasingly larger both in the quality of movement execution and in the constancy of performance [48,49]. This difference can manifest in conditioning and coordination abilities, the coordination of fine and gross motor movements, execution success, movement dexterity, and a variety of movement repertoire, and slower development has been observed in both fine and gross motor areas [49]. Physical development disorders, therefore, often lead to disharmony in motor skills [45,50,51]. The difference in motor pattern can be experienced in clumsy and uncoordinated movement execution, poor static and dynamic balance ability, weakness in head control when pulling infants into a sitting position, and asymmetrical movement execution during crawling and climbing [35,52,53,54].

Since more muscle groups are involved during the execution of movements, the movement is not economical, so the child becomes tired more quickly, which can result in a lower level of physical activity [55,56]. These studies provide evidence for the increased energy expenditure and fatigue associated with movement in individuals with ASD, which can lead to reduced physical activity levels.

Although evidence supports that children with ASD struggle with motor deficiencies from an early age, interventions should be tailored to the child’s developmental stage. For infants with ASD, early interventions often focus on sociomotor stimulation, addressing both motor and social delays simultaneously [57,58]. As children grow, interventions shift towards social play stimulation, which can help develop both motor skills and social interaction [59,60]. For older children and adolescents with ASD, sports and movement-rich activities, such as co-operative games and collective sports, offer wide opportunities for inclusion and social interaction development [61,62,63,64]. This progression of interventions acknowledges the intertwined nature of motor and social development across different ages [48]. Nevertheless, it should be emphasized that at all stages, a balanced approach addressing both motor and social skills is crucial for comprehensive development [34,35]. The manifestation of ASD characteristics varies considerably among individuals, presenting unique challenges for every child. This variability necessitates individualized approaches to provide support and intervention [65]. However, it is widely recognized that these characteristics profoundly affect socialization and various aspects of daily life. Studies show that children with ASD exhibit reduced participation levels and engage in a narrower range of activities compared to their typically developing peers and those with other developmental conditions [27,66,67,68,69]. This difference is especially noticeable in school-aged children and teenagers with ASD, with an even more pronounced decrease in community involvement [29,30,70,71].

Research by [72] emphasizes the importance of personalized interventions that account for the unique cognitive, behavioral, and sensory profiles of each individual with ASD. Furthermore, [73,74,75,76] highlight that individualized approaches can lead to more effective outcomes in areas such as social skills development and adaptive functioning.

Significant concerns arise regarding engagement in unstructured leisure activities, hobbies, post-school social interactions, and physical pursuits such as sports teams and clubs [26,27,31,32,33,68,72,73]. Significantly, adolescents with ASD typically spend 62% more time on screen-based media than on other activities, while their involvement in social interactive games or social media is minimal [33,77,78,79]. Parents express a strong desire to reduce their children’s engagement with screen-based leisure activities [29]. This concern is warranted, given that a preoccupation with screens is a clinically significant issue for many children with ASD, potentially leading to adverse effects on academic success, social interactions, and physical activity levels [77]. As children with ASD age, a decline in physical and social activity participation has been observed, coinciding with an increase in sedentary behaviors during adolescence [26,35,76,80].

While social skill deficits are commonly acknowledged in children with ASD, motor impairments are increasingly recognized as a significant concern. Research has documented challenges in both fine and gross motor skills, as well as balance and coordination difficulties [55,81]. Studies suggest that close to 79% of children with ASD experience some form of motor difficulty [42]. An analysis of retrospective video footage has revealed motor delays evident in early developmental stages [43], and recent findings indicate that the rate of motor skill acquisition in children with ASD lags behind that of their neurotypical counterparts [40].

In infant motor development, natural movement forms and gross motor movements such as rotations, crawling, climbing, sitting, standing, walking, etc., have specific milestones. We know the time frames by which children should produce these movements. If the first attempts at these movements and their repeated reproduction and the formation of general coordination patterns are delayed, or if they produced these but then the movement is missing, these signs may indicate motor deficits of autism spectrum disorder.

Motor research has also confirmed that over time, the gap in various motor skills between autistic children and those showing typical development grows significantly [48]. This difference in motor abilities can manifest in both conditional and coordination skills, as well as in the coordination pattern of fine and gross motor movements, the success of execution, motor dexterity, and the diversity of movement repertoire [34,35]. However, it can also be said that sports, various movement tasks and cooperative games offer a colorful palette of opportunities for inclusion. Despite the potential in developing motor skills, research has only recently turned towards motor intervention [64,81,82,83]. Even though motor skills are not included in the diagnostic criteria for ASD, research has demonstrated that individuals with ASD frequently exhibit deficits in both gross motor [82] and object control [83,84,85] abilities.

Research on the motor skills of children with ASD have raised the question of how motor delay or limitation affects social activities or perhaps whether motor underdevelopment causes social difficulties [81]. Since our various locomotor and non-locomotor movements, i.e., both fine and gross motor movements, are essential in the interaction of social behavior, the development of motor skills is the fundamental background for ensuring social development [86]. Research indicates that children with ASD who exhibit poorer motor skills frequently encounter more difficulties in social domains as well, finding it much more difficult to engage in joint activities and adapt to others, i.e., develop and apply social competencies, so group or social tasks in movement development, although challenging, can also develop social interaction [25,31,33]. Pusponegoro et al. [42] concluded in their research that the motor difficulties and limitations of children with ASD are behind the fact that these children have much poorer participation in activities and, in parallel, lower self-esteem.

Thanks to new screening methods used in young children, signs of ASD can be recognized as early as 12 months of age [87]. Today, most evidence-based treatments available for young children with ASD focus on addressing fundamental deficiencies in social and communication skills, as well as managing problematic stereotypical behaviors [88,89,90]. However, according to the latest research, children with ASD also experience motor developmental delays and delayed development, which appear in the early stages of life [35,43,89,91,92,93,94]. In early movement development, these delays need to be addressed, underdeveloped skills need to be improved, and the coordination of natural movement forms needs to be enhanced. Although motor skill development appears in early intervention, interventions targeting motor skills are still rare in early movement development, especially as primary developmental target areas. However, the development of motor skills is a key aspect of early childhood development that affects a child’s ability to explore their environment, interact with others, and perform daily activities. For children with autism spectrum disorders (ASDs), the challenges of motor development can be significant and multifaceted and can impact their social, cognitive, and physical growth. Understanding and supporting the motor development of children with ASD is vital for their overall development and quality of life.

There are various tests known that focus on examining motor functions in early childhood, such as the EMQ (the Early Motor Questionnaire), which measures the motor development of infants aged 0–24 months based on parental reports [95]. This questionnaire assesses development on a gross motor, fine motor, and perceptual scale, taking into account the pace of motor development. In terms of gross motor skills, it examines whether a child can sit without support, crawl forward, or maintain stability while turning their head and torso when sitting. In the area of fine motor skills, it evaluates the successful grasping of small objects, reaching for objects, and one- or two-handed grips. In the domain of motor perception, it assesses whether the child notices their hand, visually follows it, or turns their head to track a moving object [96]. Also considered effective is the Peabody Developmental Motor Scale-2 (PDMS-2), which provides an assessment of gross and fine motor functions from birth to 5 years of age [97].

The Bayley-III test is also well suited for assessing motor skills and is designed to determine the motor maturity of infants aged 1 to 42 months. This test offers the opportunity to examine children who are at high risk for ASD due to their genetic background, as the Bayley-III is also used to assess the motor profile of premature infants [98]. Beyond motor development (both fine and gross motor skills), the test evaluates other areas, such as cognitive, language (receptive and expressive), social–emotional, and adaptive behavior [99]. The Bayley-III identifies and tracks the achievement of developmental milestones, measuring aspects like bringing the hand to the mouth, grasping a moving ring, reaching and grasping with hands, reaching accuracy, laterality, and the development of elementary movement patterns, object use, and tool use [100,101].

Several studies focus on parents, as the family, being the primary context for socialization, greatly influences the cognitive, motor, emotional, and social development of both typically developing children and those with ASD. Although research focusing on parents’ experiences and perceptions regarding their children’s development has been present for some time [102,103,104], recent years have seen a renewed interest in this area [105,106,107,108]. Several studies report that parents may play a significant role in early diagnosis as they are often the first to notice developmental delays or issues in motor, social, or cognitive functions [109]. Other research highlights that a supportive parental environment and active parental involvement with the school can promote the academic progress and development of various skills in children with ASD [108]. Another study indicates that parents diagnosed with ASD and ADHD, who experience stress related to their child’s diagnosis and are more stressed in general, negatively impact their children’s ability to develop their skills [110]. Research declares that parent training leads to significant improvements in both the parents’ ability to implement interventions and in the communication skills of children with ASD. This highlights the effectiveness of these interventions in fostering critical communication skills in children, a core challenge faced by individuals with ASD. This research underscores the value of involving parents in intervention processes for children with ASD, enhancing communication skills, and pointing to areas where further investigation is needed to optimize training methods [111].

## 2. Materials and Methods

### 2.1. Study Aim and Participants

The aim of this study is to explore parental perspectives, challenges, and strategies related to movement development in 3–10-year-old children living with autism. For our research, we used semi-structured in-depth interviews, which allowed participants to share their experiences, feelings, and strategies related to their child’s movement development in detail. During the interviews, we interviewed 51 parents raising a child with autism).

#### 2.1.1. Selection of Participants

Participants were widely selected from those who participated in early movement development provided by ÉFOÉSZ (the National Organization of People with Intellectual Disabilities). We ensured national coverage, with respondents from all counties in Hungary. Counties with a lower population were represented by two participants, while counties with higher population numbers were represented by three participants. In sampling, we selected families with different backgrounds to gather the widest possible range of experiences. An important criterion in selecting parents was that their child should be in the 3–10 age group within the autism spectrum. Only parents of children with a medical diagnosis certificate of ASD and documentation of the diagnosis time were included in the sample.

#### 2.1.2. Conducting Interviews

The semi-structured interviews allowed us to handle conversations flexibly while ensuring that all relevant topics were covered. During the interviews, we used a predetermined set of questions that guided the conversation but also allowed participants to freely express their own experiences, thoughts, and feelings. Interviews were conducted in person, by phone, or via an online video call, taking into account the preferences of the given family and pandemic restrictions. Each interview lasted 45–60 min, with an average duration of 52 min.

#### 2.1.3. Interview Questions

When designing the interviews, special attention was paid to ensure that the questions were sensitive to the special needs of families and children living with autism. The questions covered the following topics:

1. Basic information about the child, including the time and circumstances of the autism diagnosis.
2. Current movement development activities and challenges perceived by the parent.
3. Types of movement enjoyed by the child and factors influencing motivation.
4. The impact of movement development on the child’s communication and social interactions.
5. Support and resources available in the family and home environment.
6. The child’s unique needs and planning of the movement development program.
7. Goals set by the family and the help needed to achieve them.
8. Steps planned for the near future and special requests to professionals.

The specific interview questions can be found in the Appendix A.

#### 2.1.4. Data Processing Method

The audio recordings of the interviews were transcribed, and then the textual data were subjected to content analysis. During content analysis, we created codes and categories to structure the data and identify key themes. The grouping of responses received by the questions and their key categories can be seen in the result section.

In analyzing the data, we first applied the open coding method, which allowed us to discover new themes and connections in the data. We then applied selective coding to organize information around the main themes, thus ensuring an in-depth and comprehensive analysis of the data.

During data analysis, we paid special attention to ethical considerations, ensuring that participants’ personal data were handled confidentially and the data were anonymized. We removed all personal information and identifiers that emerged during the analysis to fully preserve the anonymity of the participants. Throughout the research, we obtained consent from all participants, informing them about the purpose of the research, its methodology, and how the data would be handled. We ensured the anonymity of the participants and the confidential handling of their data.

We thoroughly examined the interview transcripts and, using the open coding method, identified the most common themes and patterns relevant to the research objectives. We analyzed the data thematically to understand the challenges, concerns, and successful strategies articulated by the parents. To create the table, we first present the most common themes and key categories identified during open and selective coding and then organized these and presented them in a tabular form. The table shows the results of 51 parent interviews.

#### 2.1.5. Sample

Table 1 and Table 2 describe the socioeconomic background of the parents. The tables show the type of residence, number of children raised, education level of the mother and father, net income per capita, and the proportion of single mothers raising their children.

For parents raising children with ASD, the average age of mothers was 36.62 years (SD = 5.93), with the youngest mother being 23 and the oldest 50 years old. For fathers, the average age was 40.76 years (SD = 6.35), with the youngest father being 29 and the oldest 54 years old. The decimal age of children diagnosed with ASD ranged from 3.12 to 10.11 years (37 months to 121 months). The earliest diagnosed children were detected at 24 months, while the latest diagnosed were 58 months of age.

## 3. Results

The data and theme categories were selected as a result of open and selective coding, which helped identify the most common concerns, challenges, and strategies related to movement development in children living with autism (Table 3).

### 3.1. Movement Development Challenges

Parents reported delayed motor development, a poor quality of movement execution, uneven development, and a lack of coordination. These delays were observed in activities such as using eating utensils, tying shoelaces and buttoning clothes, and grasping and reaching for building blocks and other objects. Uncertainty in walking, jumping, and balancing was also frequently mentioned. These parental observations align well with standardized assessments like the Bayley-III test and other studies on parental perception [93,98,103,107]. Among the challenges related to movement development for autistic preschool children, communication barriers and difficulties in group interactions (when children need to play together, engage in sports, adapt to each other, and communicate with each other) were mentioned by many, which align with experiences from other international studies that have also confirmed this. Parents highlighted that children often struggle to express their movement needs, leading to misunderstandings and frustrations. One parent shared, “It’s often difficult to understand what he wants. For example, if he’s tired or hungry, he communicates more easily, but when he wants to move, he just gestures or becomes agitated.” (interviewer 12). This variability in communication underscores the complex nature of understanding and addressing these needs. Similar difficulties were identified by Duquette et al. in their 2016 study of young people [113].

Social interaction in group activities posed a challenge for many children, as adapting to peers and the presence of other children often interferes with engaging in the given sports activity or the activity level. One parent noted, “Group activities (a joint game of several children) are difficult for him. You can see he wants to participate, but when there are too many children around him, he just withdraws.” (interviewer 4) This highlights a significant barrier to participation in large group-based movement activities, which are often recommended for social skill development [132]. To address this barrier, Zhao and Chen suggest structured physical activity programs, which they found significantly improved communication, cooperation, and social interaction in children with ASD [133]. Children with autism spectrum disorder (ASD) are unique not only in their diagnosis but also in their interests and motor abilities, presenting a significant challenge for parents. They must find the most individually motivating movement programs for their children, as reported by several parents. “The biggest challenge was finding the right activity that was interesting enough for him but didn’t cause overload due to his sensory sensitivity.” (interviewer 3). Another parent shared, “Sometimes it was difficult to motivate him to participate in movement development exercises, especially on bad days. But when we found the right motivational factors, it made a huge difference.” (interviewer 32). Jin et al. found in their study that children who found their physical education class interesting/exciting participated more actively [116]. These anecdotes illustrate the delicate balance parents must achieve between stimulating interest and avoiding sensory overload.

### 3.2. Enjoyable Forms of Movement

Based on parents’ responses, it can be concluded that in many cases, parents try to involve children in other movement activities beyond early movement development, either through organized movement programs or through joint sports activities with the family or parent. This included various water programs, animal-assisted therapies such as dog and horse therapy, various ball movement tasks, a mini-trampoline, sensory games, playground activities encouraging gross motor skills (the use of slides, spinners, and climbing equipment), music and dance movements, TSMT and other physiotherapies, and movements performed on obstacle courses. This diverse array of activities underscores the necessity of tailoring movement programs to each child’s unique preferences and capabilities. Yanardağ and Yilmaz mentioned similar activities in their study [118]. Parent interviews also support that when a parent finds movement that provides movement development for the child that they love, they perform it with great joy and activity. One parent observed, “Swimming works wonders for him. When he’s in the water, he completely calms down, and you can see he enjoys it.” (interviewer 1). The calming effect of water and the enjoyment derived from swimming highlight the potential benefits of incorporating aquatic activities into movement programs. In addition to the motivating role of water, music appeared to be highly effective for many children. “Music somehow reaches him. When we listen to music and dance, he completely blossoms. He’s much more open and active then.” (interviewer 5). This suggests that combining music with movement might enhance engagement and enjoyment for children with ASD. Koch et al. found in their document analysis study that imitation exercises often used in dance helped develop social skills in children with ASD [120].

Another parent reported that in movement development classes, their child really liked natural movements performed on various obstacle courses, balance development, and sensory tasks, so they often carry out these tasks adapted to the home environment. “At first, I was also worried about how my child would accept these activities, especially because of his autism. But then I realized that the key lies in sparking his interest. With the obstacle course, for example, I noticed that my child was very interested in different textures and shapes. So I decided to use this interest to motivate him to move. We started building smaller obstacle courses at home, using everything we could find: pillows, boxes, and other objects that evoked different feelings in him. I observed how much he enjoyed the new challenges, and as he became more skilled, his self-confidence grew as well.” (interviewer 7). This example highlights the importance of creating stimulating and varied environments that cater to a child’s sensory interests and promote motor skill development.

Animal-assisted activities also emerged as a beneficial approach. These activities help children release their inhibitions and improve their contact and social–communication skills, which international studies have confirmed [134,135]. One parent highlighted the motivating role of dog activities in encouraging movement. “Regarding the dog activities, I thought that my child’s natural love and curiosity towards animals could be the point of connection. The direct and non-judgmental love of dogs had a wonderful effect on him. At first, we just observed, then slowly involved him in interactive games and exercises. In the company of dogs, he unconsciously became more active and open. The most important thing was to always let my child set the pace. We never forced anything on him but let him discover for himself what activities brought him joy. And as he opened up to these, we also made the tasks more varied and challenging.” (interviewer 21). This narrative underscores the potential for animal-assisted activities to provide a natural and enjoyable way for children to engage in movement. A 2019 study reported similarly good effects of animal-assisted activities, which can improve the condition of people with mental or physical health issues [136].

### 3.3. Motivational Factors

Regarding new forms of movement and movement development, parents also reported that with motivation, which can either take the form of small gifts or active parental participation, children are much more motivated to try new things or continuously practice already known forms of movement. “If he knows he’ll get some positive feedback, he’s much more willing to try new things. Praise and stickers motivate him a lot.” (interviewer 42). The use of rewards as a motivational tool aligns with behavioral approaches commonly used in therapy for children with ASD. Parental participation was also highlighted as a significant motivational factor. “If we do the exercises together, he’s much more enthusiastic. He feels this is our shared time, and it’s very important to him.” (interviewer 14). This indicates that movement activities can serve as valuable bonding experiences between parents and children, enhancing motivation and participation.

In movement development and skill development, a supportive environment plays a very important role. Parents highlight the helpfulness of professionals, therapists, and physiotherapists and the provision of personalized tasks adapted to individual needs, which ensure development for the child. The other area mentioned in the interviews is family support. If parents, grandparents, and siblings help not only with the difficulties of everyday life but also in movement development, either through home practice or trying new activities, this supportive home environment positively manifests in the results of movement development. Ryan-Lewis [125] also confirms in their study that parental participation helped improve their child’s language and communication skills. This holistic support system is crucial for consistent progress and development in children with ASD.

### 3.4. Special Needs and Interventions

Since children diagnosed with autism spectrum disorder are very different, with differences that can manifest in cognitive, motor, and social skills alike, the application of personalized and individual interventions in the development of the motor area is very important. This personalized movement development, as an expectation, is supported by parent interviews due to the observed differences in preferred forms of movement or experiences of different challenges related to movement development. They found that personalized movement therapy was more effective than general therapy. “Every child is different. An individual therapy plan tailored to their own needs is much more effective than general programs.” (interviewer 8). This underscores the necessity of creating bespoke movement programs that cater to the specific needs and preferences of each child.

Although our research focused on movement development, parents prominently displayed not only the development of motor skills (coordination, balance, and conditional abilities) among further development goals but also the development of communication skills and social or interpersonal skills. Parents’ expectation and goal is that these skills will also develop through movement activities and group movement tasks. It is no coincidence that parents’ plans include further strengthening participation in group activities and trying new therapeutic methods that develop the child’s abilities by focusing on the connection between movement and communication.

### 3.5. Goals and Expectations

We also examined how parents’ goals develop during the interventions, where the development of communication skills and social interactions clearly received increased focus as the most noticeable disadvantage can be observed in this area. However, parents’ responses also revealed that group movement therapy sessions or movement therapy can help achieve these goals. We highlighted the following from parents’ responses: “Our next big goal is for him to participate more confidently in group sports activities. We want him to learn to enjoy teamwork and shared successes.” (interviewer 19). Another parent added, “We want to continue the sensory integration exercises so he can better handle various stimuli. The goal is for him to feel more comfortable in diverse environments.” (interviewer 25).

We were curious about the responses related to parents’ future plans. “Our next big goal is for him to participate more confidently in group sports activities. We want him to learn to enjoy teamwork and shared successes.” (interviewer 8). The parents’ responses yielded the result that they plan to involve additional professionals to improve the success of the therapy and prefer group activities. This holistic view of movement therapy’s benefits aligns with emerging research on the interconnectedness of motor, social, and cognitive development in ASD. It suggests that movement-based interventions could have far-reaching effects beyond physical development.

## 4. Discussion

A notable trend in the findings was the growing interest in group activities and social participation. While many parents reported initial difficulties with group settings, there was a clear desire to increase their child’s involvement in social movement activities. This indicates a shift towards more holistic developmental goals and the recognition of the social benefits of movement therapy. Similar trends have been observed in previous studies, such as that by [93], who found that parents of children with ASD increasingly value social engagement opportunities in movement-based interventions. These findings align with those of [94], who developed the Early Motor Questionnaire (EMQ) to assess parents’ perception of motor development in children with ASD.

This study provides valuable insights into parental perspectives on movement development for children with autism spectrum disorder (ASD). Communication barriers and social interaction difficulties emerged as significant challenges, underscoring the need for personalized approaches to movement therapy. Motivational factors, including small rewards and parental participation, were crucial for engaging children in movement activities, as confirmed by other research [133]. This emphasis on motivation echoes the findings of [130], who stressed the importance of engaging, motivating activities in motor interventions for children with ASD [106].

The integration of music, water activities, and animal-assisted therapies offered promising avenues for movement engagement, resonating with many children. This aligns with the work of [137], who found that music-based interventions can significantly enhance motor and social skills in children with ASD. Parents recognized the potential of movement development to improve motor, communication, and social skills, highlighting the interconnectedness of these developmental areas. Parental observations can be valuable in both diagnosing and developing interventions for children with ASD, as confirmed by other studies [103,104,120,121,122].

The involvement of professionals and family support systems was crucial for the success of movement programs, suggesting a need for collaborative approaches bridging clinical and home environments. This finding is consistent with the research of [39], who emphasized the importance of parent-mediated interventions in ASD treatment. Tailored, multifaceted approaches incorporating individual and group activities are essential for effective movement development in children with ASD. A study similarly reports that young adults with ASD were more active in physical activities and sports where parental support was high, meaning parents encourage their children to be physically active, take them to places where they can be active, and have the financial means to join health clubs [138]. Future research should explore the long-term impacts of personalized movement interventions and develop training programs for parents and caregivers. This aligns with recommendations by [135], who advocate for more longitudinal studies on ASD interventions. Integrating technology and innovative approaches like animal-assisted therapy into movement programs warrants further exploration, which has been confirmed in other research [135,136]. This study emphasizes the importance of individualized, family-centered approaches, unlocking new pathways for improving the overall well-being and functioning of children with ASD.

This holistic perspective on movement development aligns with broader therapeutic goals, where the enhancement of motor skills is seen as a gateway to broader improvements in communication and social interaction. This interconnected view of development is supported by the work of [129], who found strong associations between motor and social skills in children with ASD [137,138,139].

Parents’ observations about their children’s positive responses to tailored movement activities suggest that such programs can lead to significant developmental gains. This finding is consistent with the research of [58], who found that parent-reported outcomes are valuable in assessing the effectiveness of sensory–motor interventions for children with ASD.

This study also highlights the crucial role of environmental and contextual factors in supporting movement development. The support from family members and the inclusion of professional guidance were repeatedly mentioned as pivotal. These factors underline the necessity for a comprehensive support system that integrates home-based activities with professional therapeutic interventions, a conclusion also reached by [140] in their review of home-based interventions for ASD.

Importantly, the diversity of enjoyable forms of movement reported by parents underscores the necessity for flexible and adaptable movement programs. From swimming to music and dance, the range of activities that can engage children with ASD is vast, and programs must be prepared to cater to these varied interests. This finding is supported by the work of [141], who found that a variety of physical activities can benefit children with ASD.

The motivational aspect is particularly noteworthy, as it bridges the gap between the child’s natural interests and structured therapeutic activities. Whether through small rewards or parental involvement, motivation is a key driver of engagement. This insight calls for movement programs that are not only scientifically grounded but also creatively designed to sustain the child’s interest and enthusiasm, a principle also emphasized by [142] in their research on pivotal response treatment for ASD.

## 5. Conclusions

This study offers a significant contribution to our understanding of movement development in children with autism spectrum disorder (ASD). It highlights the intricate interplay between motor skills, social interaction, and cognitive development, underscoring the critical need for a paradigm shift in intervention strategies.

Our findings emphatically demonstrate the necessity for highly personalized, engaging, and supportive movement programs that are tailored to address the unique needs of each child with ASD. By elucidating the profound interconnectedness of motor, social, and cognitive domains, this research opens up promising new avenues for holistic and more effective intervention strategies.

This study’s results advocate strongly for a collaborative, multidisciplinary approach that seamlessly integrates the efforts of parents, healthcare professionals, educators, and the broader family environment. This synergistic approach has the potential to enhance the overall quality of life for children with ASD, fostering improvements not just in motor skills but in communication, social interaction, and cognitive functioning.

Moreover, our research underscores the transformative power of motivation and engagement in movement-based interventions. By leveraging each child’s unique interests and strengths, and by involving parents actively in the therapeutic process, we can create more effective, sustainable, and enjoyable interventions that yield long-term benefits.

In conclusion, this study serves as a call for a more complex, individualized, and holistic approach to ASD intervention. It challenges the traditional boundaries between different developmental domains and argues for an integrated perspective that recognizes the child as a whole. As we move forward, it is imperative that researchers, clinicians, and policymakers heed these insights to develop more comprehensive, effective, and compassionate strategies for supporting children with ASD in reaching their full potential.

### Limitations

This study’s sample size and diversity, while substantial for qualitative research with 51 parent interviews, may not be fully representative of all families with children with ASD. The focus on Hungary limits the generalizability of findings to other cultural contexts. The age range of 3–10 years excludes perspectives on adolescents and younger toddlers with ASD, whose challenges and needs may differ significantly.

The reliance solely on parental reports provides a valuable but limited perspective. Including insights from children with ASD themselves (where possible), therapists, and educators could offer a more comprehensive view of movement development challenges and strategies. The lack of longitudinal data means this study provides only a snapshot of the current perspectives, without tracking changes over time.

There is potential for bias in the sample as parents who agreed to participate might be more engaged in their child’s movement development, potentially skewing results towards more positive or proactive perspectives. This study did not extensively explore how the severity of ASD symptoms might influence movement development challenges and strategies, which could be an important factor in tailoring interventions.

While the qualitative approach provided rich, detailed information, the lack of quantitative data limits the ability to make statistical inferences or comparisons. The semi-structured nature of the interviews, while allowing for flexibility, might have introduced some inconsistency in the data collection process.

These limitations suggest opportunities for future research to address these gaps by including a larger, more diverse sample, incorporating multiple perspectives, conducting longitudinal studies, and combining qualitative with quantitative methods to provide a more comprehensive understanding of movement development in children with ASD.

## Figures and Tables

**Table 1:** Distribution of parents’ sociodemographic data.

Demography	Category	Distribution
Residence	Village	26.00%
	City	48.00%
	County seat	26.00%
Number of Children	1	38.00%
	2	38.00%
	3	16.00%
	4	8.00%
Mother’s education	Elementary school	14.00%
	Vocational school	18.00%
	High school	46.00%
	University	22.00%
Father’s education	Elementary school	4.00%
	Vocational school	26.00%
	High school	44.00%
	University	26.00%
Single parent	Yes	36.00%
	No	64.00%
Net monthly income per capita	Less than EUR 405	20.00%
	EUR 405–540	32.00%
	EUR 541–675	30.00%
	EUR 676–810	10.00%
	More than EUR 810	8.00%

**Table 2:** Mean and standard deviation values of families’ sociodemographic data.

Demography	Mean	Standard Deviation
Mother’s age	36.62 years	5.93
Father’s age	40.76 years	6.35
Net monthly income per capita	EUR 557	60,386
Age of child with ASD (decimal age)	6.99	2.13
Time of diagnosis (month)	37.66	8.22

**Table 3:** Parental views on movement development for children with autism.

Topics/Key Categories	Frequency (n = 51)	Notes Based on Parental Responses	Experiences from Previous Research Supporting Parents’ Statements
Family Structure			
Dual-Parent Household	33		NA
Single Parent	18		NA
Foster Parent	25		NA
Developmental Movement Challenges			
Communication Barriers	32	Children have difficulty expressing their need for movement	[112]
Social Interaction	29	Difficulty integrating into group activities	[113]
Motivation	37	Finding a preferred form of movement	[114,115,116]
Enjoyable Forms of Movement			
Individual sports	26	E.g., swimming, horseback riding	[117]
Combination of Music and Movement	37	Dance, rhythmic gymnastics	[118,119,120]
Motivational Factors			
Rewarding	41	Positive feedback, small rewards	[121]
Parental Involvement	48	Children are more motivated when parents are involved	[122,123,124]
Support and Resources			
Professional Support	45	Involvement of therapists, special educators	
Family Support System	38	Help from siblings, grandparents	[125]
Special Needs and Interventions			
Personalized Programs	49	Necessity of an individual therapeutic plan	[126]
Sensory Stimulation	44	Use of sensory toys, tools	[74,127]
Goals and Expectations			
Developing Communication Skills	35	Speech and non-verbal communication	[128]
Improving Social Skills	40	Interaction with other children	[58,59]
Next Steps			
Involving More Specialists	27	Trying new therapeutic methods	[129]
Group Activities	34	Increasing participation in social environments	[130,131]

## Data Availability

Data will be made available on request.

## Appendix A

**Sociodemographic requests**
The age, educational background, place of residence, per capita income, number of children of the parents (father, mother) raising the child, birth date of the child with ASD, and the child’s age at the time of diagnosis (given in months). Family circumstances (divorced, single-parenting, foster parent).
**Interview Questions:**

1. **Basic information about the child**
  When and under what circumstances did you receive your child’s ASD diagnosis?
  What were the precedents for the diagnosis? Was there any assessment or evaluation conducted?

2. **Current movement development activities and challenges**
  What movement development activities are you currently engaging in with your child?
  What challenges do you experience during these activities?

3. **Types of movement enjoyed by the child and motivational factors**
  What types of movement are your child’s favorites?
  What factors influence your child’s motivation for movement?

4. **The impact of movement development on the child’s communication and social interactions**
  How has movement development impacted your child’s communication skills?
  How has your child’s social interaction changed as a result of movement development?

5. **Support and resources available in the family and home environment**
  What support and resources are available at home for your child’s development?
  Is there any organization or community that you have turned to for assistance?

6. **The child’s unique needs and planning of the movement development program**
  What unique needs do you observe in your child regarding movement development?
  How do you plan the movement development program to meet these needs?

7. **Goals set by the family and the help needed to achieve them**
  What goals have you set regarding your child’s movement development?
  What help do you need to achieve these goals?

8. **Steps planned for the near future and special requests to professionals**
  What steps do you plan to take in the near future regarding your child’s movement development?
  Do you have any special requests for professionals that could help in your child’s development?
